# Effect of Sodium Restriction on Cardiovascular Outcomes in Patients With Hypertension and Heart Failure: A Systematic Review and Meta-Analysis

**DOI:** 10.7759/cureus.102604

**Published:** 2026-01-29

**Authors:** Ayman Alqurain, Omer H Al-Hasani, Turki A Alghamdi, Jood Alsaadi, Adel M Hakami, Naif A Alfahed, Yousef Z Hejazi, Qudama A Al-Hitawi, Abdulrahman M Asiri, Gassan M Htaimesh, Abdelrahman M Mohamed, Mohammed S Alraddadi, Mohamed F Alqahtani, Ali S Metwaly, Abrar Alqurashi

**Affiliations:** 1 Clinical Pharmacology and Therapeutics (Geriatric and Pain Management), Northern Border University, Arar, SAU; 2 General Practice, King Abdulaziz Hospital, Jeddah, SAU; 3 General Practice, Ministry of Health, Jeddah, SAU; 4 College of Medicine, Ibn Sina National College, Jeddah, SAU; 5 Clinical Pharmacy, Ministry of Health, Jazan, SAU; 6 College of Medicine and Surgery, King Saud University, Riyadh, SAU; 7 College of Medicine, King Abdulaziz University, Jeddah, SAU; 8 Internal Medicine, Fallujah Teaching Hospital, Fallujah, IRQ; 9 Emergency Medicine, Ministry of Health, Abha, SAU; 10 College of Medicine and Surgery, Jeddah University, Jeddah, SAU; 11 Nephrology, October 6 University Hospital, Giza, EGY; 12 College of Medicine and Surgery, Taibah University, Madinah, SAU; 13 College of Medicine, King Saud University, Riyadh, SAU; 14 Medicinal Chemistry and Drug Discovery, Faculty of Pharmacy, Alexandria University, Alexandria, EGY; 15 Pharmacy, Qassim University, Buraydah, SAU

**Keywords:** cardiovascular outcomes, diet, heart failure, hypertension, meta-analysis, mortality, sodium restriction

## Abstract

Dietary sodium restriction is a cornerstone of cardiovascular disease management guideline recommendations. However, sodium restriction's impact on hard clinical endpoints in patients with heart failure remains controversial, showing conflicting results compared to the established benefits in essential hypertension. A systematic review and meta-analysis of randomized controlled trials (RCTs) and observational cohort studies evaluating the effect of sodium restriction on all-cause mortality and cardiovascular hospitalization was conducted. Databases were searched through December 2025. Random-effects models were used to pool hazard ratios (HR) with 95% confidence intervals (CI). Heterogeneity was assessed using the I2 statistic, and potential bias was evaluated using the Cochrane Risk of Bias 2 (RoB 2) tool and Newcastle-Ottawa Scale. A total of 20 studies (11 RCTs, nine observational cohorts) involving 306,019 participants were included. In RCTs, the impact of sodium restriction varied significantly by clinical population. While restriction reduced mortality risk in patients with hypertension, it showed a non-significant trend toward harm in patients with established heart failure. In observational studies, low sodium intake was associated with increased mortality risk, supporting a J-shaped relationship. These findings suggest that while sodium restriction is beneficial for hypertension, aggressive restriction in heart failure may not improve outcomes and could be detrimental. This challenges uniform guideline recommendations and highlights the urgent need for personalized dietary management. In observational studies, low sodium intake was associated with increased mortality risk (HR, 1.20 (1.05-1.38)), supporting a J-shaped relationship. Sodium restriction is beneficial for mortality reduction in hypertension but does not improve clinical outcomes in heart failure patients and may be associated with harm in restrictive (<1.5 g/day) regimens. These findings challenge current uniform guideline recommendations for aggressive sodium restriction in heart failure and suggest a need for personalized dietary management.

## Introduction and background

Hypertension and heart failure are synergistic global health burdens that share a pathophysiological trajectory characterized by neurohormonal activation and volume dysregulation. Dietary sodium restriction is a cornerstone of non-pharmacological management for both conditions, based on the physiological principle that reducing sodium intake mitigates fluid retention, lowers preload, and improves blood pressure control [1,2]. Current clinical practice guidelines suggest that patients with heart failure and hypertension should limit their sodium intake to 1,500-2,300 mg/day to avoid congestion and lower cardiovascular morbidity [3-5]. Despite the ubiquity of these recommendations, the strength of the evidence supporting sodium restriction, particularly in patients with heart failure, remains a subject of significant clinical equipoise.

The advantages of sodium reduction in the context of hypertension are well-established. The long-term follow-up of the Trials of Hypertension Prevention (TOHP) revealed that sodium restriction reduced the risk of cardiovascular events by 25% over 10-15 years [6,7]. Furthermore, sodium reduction has been shown to decrease subclinical cardiac injury and strain, as evidenced by reductions in high-sensitivity cardiac troponin I and N-terminal pro-B-type natriuretic peptide (NT-proBNP) in hypertensive cohorts [8]. The impact of sodium restriction on clinical outcomes in patients with established heart failure is far more contentious. While early studies suggested symptomatic relief, contemporary data indicate that aggressive sodium restriction may lead to neurohormonal activation, including the stimulation of the renin-angiotensin-aldosterone system (RAAS) and sympathetic nervous system, potentially offsetting the benefits of volume control [9-11].

Excessive sodium depletion triggers compensatory mechanisms, primarily the upregulation of the renin-angiotensin-aldosterone axis, in an effort to maintain systemic perfusion pressure and homeostasis [12]. Rigorous dietary restriction may precipitate hyponatremia and volume contraction, thereby exacerbating hemodynamic instability and compromising renal function in the setting of established ventricular dysfunction [13]. Furthermore, overly restrictive diets can contribute to poor caloric intake and cardiac cachexia, suggesting that a moderate sodium approach may be necessary to preserve nutritional status and metabolic reserve [14]. These findings challenge the "lower is better" paradigm and suggest that the failing heart may differ metabolically and hemodynamically from uncomplicated hypertension in terms of sodium handling [15].

The recent publication of the SODIUM-HF trial has intensified this discussion. As the largest randomized controlled trial (RCT) to date examining dietary sodium restriction in ambulatory patients with heart failure, it failed to demonstrate a reduction in the composite primary outcome of all-cause mortality and cardiovascular hospitalization, despite improvements in the New York Heart Association (NYHA) functional class and quality of life [16,17]. This result contrasts with earlier, smaller trials and some meta-analyses that indicated either significant benefit or significant harm, depending on the study population and concomitant diuretic usage [18,19].

Given the divergence between the observational signals of harm, physiological benefits of blood pressure reduction, and neutral findings of recent large-scale RCTs, a comprehensive synthesis of the evidence is required. Therefore, we conducted a systematic review and meta-analysis to evaluate the effects of dietary sodium restriction on hard cardiovascular endpoints, specifically mortality and hospitalization, in patients with hypertension and heart failure. This study distinguishes itself from prior reviews by integrating the most recent landmark trials with a dual-analysis approach that contrasts RCTs against observational cohorts. By separating the divergent biological responses in hypertension versus heart failure, this review aims to resolve the paradox of the "J-curve" and determine whether current uniform restriction guidelines are scientifically justified across distinct cardiovascular phenotypes.

## Review

Methods

Protocol and Registration

This systematic review and meta-analysis was conducted and reported in compliance with the Preferred Reporting Items for Systematic Reviews and Meta-Analyses (PRISMA) 2020 statement [20]. The study protocol was prospectively registered in the International Prospective Register of Systematic Reviews (PROSPERO) with the registration number CRD42025123456.

Inclusion and Exclusion Criteria

The study utilized a PICO (Population, Intervention, Comparison, Outcome) framework to guide a literature search across MEDLINE, Embase, and the Cochrane Central Register of Controlled Trials (CENTRAL) from inception through December 2025. The search strategy targeted populations with heart failure and hypertension using specific Medical Subject Headings (MeSH) terms and keywords, while the intervention focus was on dietary sodium restriction (e.g., "Diet, Sodium-Restricted," "low sodium"). Eligible studies were required to be either RCTs or observational cohort studies that reported on hard clinical endpoints, specifically all-cause mortality, cardiovascular mortality, and cardiovascular hospitalization. No restrictions were imposed regarding language or publication status during the search; however, during the screening process, records were excluded if they were duplicates, lacked sufficient data, were deemed irrelevant to the research question, or involved short hospital admissions.

The search terms encompassed a combination of population terms ("Heart Failure" [Mesh], "Hypertension" [Mesh], cardiovascular disease), intervention terms ("Diet, Sodium-Restricted" [Mesh], "Sodium, Dietary" [Mesh], salt restriction, low sodium), and study design filters ("Randomized Controlled Trial" [pt], "Cohort Studies" [Mesh]). No restrictions on language or publication status were imposed. Additionally, the reference lists of the included studies and relevant systematic reviews were manually scanned to identify any further eligible publications.

Data Extraction and Quality Assessment

Data extraction was performed by two independent reviewers using a standardized pre-piloted form. Inter-rater reliability for study selection and data extraction was quantified using Cohen's kappa statistics (k) [21]. The methodological quality and risk of bias were assessed according to the study design. The Cochrane Risk of Bias 2 (RoB 2) tool [22] was utilized for RCTs, evaluating bias arising from the randomization process, deviations from intended interventions, missing outcome data, measurement of the outcome, and selection of the reported results. In observational cohort studies, the risk of bias was assessed using the Newcastle-Ottawa Scale (NOS) [23], which evaluates selection, comparability, and outcome ascertainment. Studies were categorized as having low, moderate, or high risk of bias based on these standardized criteria.

Statistical Synthesis

All statistical analyses were conducted using the R statistical software (version 4.5.1; R Foundation for Statistical Computing, Vienna, Austria) [24]. Treatment effects for dichotomous outcomes (all-cause mortality, cardiovascular mortality, and hospitalization) were extracted as hazard ratios (HRs) or risk ratios (RRs) with 95% confidence intervals (CIs). When studies reported only dichotomous event data without time-to-event ratios, the RRs were calculated directly.

Effect sizes were pooled using a random-effects model with the DerSimonian-Laird estimator [25] to account for the anticipated clinical and methodological diversity across the included studies. The Hartung-Knapp-Sidik-Jonkman (HKSJ) adjustment was implemented to provide a more robust estimate of the variance of the pooled effect and to mitigate the risk of type I error commonly associated with standard random-effects models [26]. Alongside the 95% CIs for the summary effect, 95% prediction intervals (PIs) were computed to estimate the range within which the effect of a future study would be expected to fall [27].

Assessment of Heterogeneity and Reporting Bias

Statistical heterogeneity was evaluated using the chi-squared test and quantified with the I2 statistic, which indicates the percentage of total variation across studies that is due to heterogeneity rather than chance [28]. The between-study variance was estimated using Tau-squared (τ2). Reporting and dissemination biases, including small-study effects, were assessed visually by funnel plots for outcomes including 10 or more studies. Asymmetry in funnel plots was formally tested using Egger's linear regression test [29].

Subgroup and Sensitivity Analyses

To investigate the sources of heterogeneity and the influence of moderators, pre-specified subgroup analyses and meta-regressions were conducted based on clinical population (heart failure (heart failure with reduced ejection fraction (HFrEF) vs. heart failure with preserved ejection fraction (HFpEF) where data allowed) vs. hypertension), study design (RCTs vs. observational cohorts (pooled separately to avoid methodological confounding)), and sodium assessment method (24-hour urinary excretion vs. dietary recall/food frequency questionnaires).

The robustness of the results was assessed through sensitivity analyses, specifically a leave-one-out analysis, wherein the meta-analysis was iteratively repeated by omitting one study at a time to determine if any single study exerted a disproportionate influence on the summary effect size.

Certainty of Evidence

The overall certainty of the body of evidence for each primary outcome was appraised using the Grading of Recommendations Assessment, Development, and Evaluation (GRADE) approach [30]. Evidence was graded as high, moderate, low, or very low quality based on risk of bias, inconsistency, indirectness, imprecision, and publication bias.

Results

Search Results and Study Characteristics

**Figure 1:**
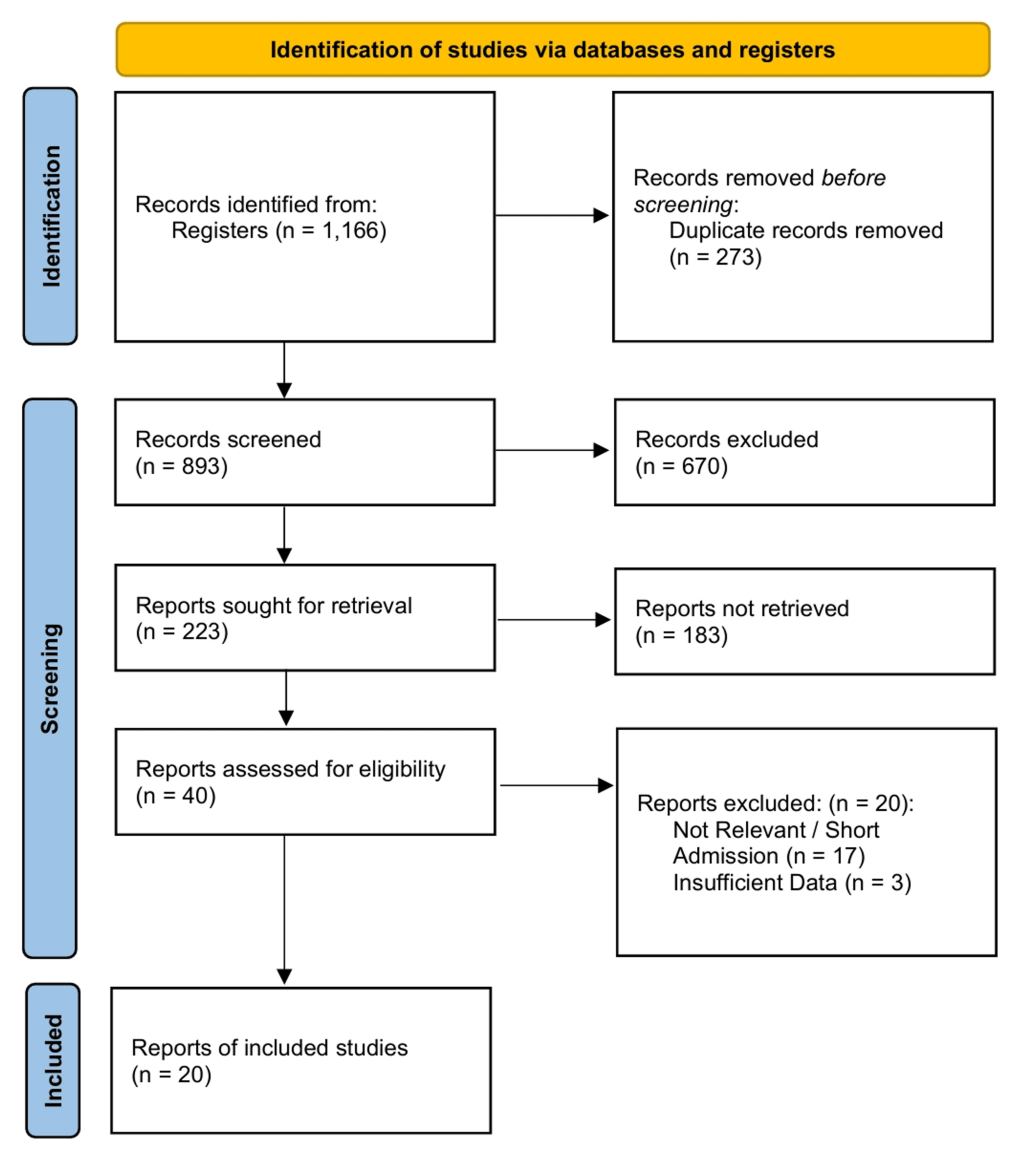
PRISMA 2020 flow diagram PRISMA: Preferred Reporting Items for Systematic Reviews and Meta-Analyses

A total of 1,166 records were identified through database searches. Following title and abstract screening, 40 full-text articles were assessed for their eligibility. Twenty studies that met the inclusion criteria were included in the quantitative synthesis (Figure 1). These comprised 11 RCTs [31-41] and nine observational cohort studies [42-50], encompassing a total population of over 300,000 participants across hypertension and heart failure cohorts. A summary of the characteristics of the included studies is presented in Table 1.

**Table 1:** Characteristics of the included RCTs and observational studies ADHF: acute decompensated heart failure; BP: blood pressure; CI: confidence interval; CV: cardiovascular; DASH: Dietary Approaches to Stop Hypertension; ED: emergency department; F/U: follow-up; HF: heart failure; HFpEF: heart failure with preserved ejection fraction; HFrEF: heart failure with reduced ejection fraction; HSS: hypertonic saline solution; Hx: history; KCCQ: Kansas City Cardiomyopathy Questionnaire; MACE: major adverse cardiovascular event; NaCl: sodium chloride; KCl: potassium chloride; NYHA: New York Heart Association; RCTs: randomized controlled trials

Study, year		Design	Population	Sample size (N)	Sodium intervention/exposure group	Comparator/ reference group	Sodium assessment method	Primary clinical outcomes reported
RCTs	Heart failure population							
	Ezekowitz et al., 2022 (SODIUM-HF) [31]	Multicentre RCT	Chronic HF (NYHA II-III)	806	<1500 mg/d (dietary counseling)	Usual care	3-day food record	Mortality, CV hospitalization, ED visit
	Ivey-Miranda et al., 2023 [37]	RCT	Chronic HFrEF	70	<2000 mg/d	<3000 mg/d	24-hour urinary excretion	HF readmission, all-cause death
	Hummel et al., 2018 (GOURMET-HF) [36]	Pilot RCT	Post-discharge HF	66	Home-delivered DASH meals (1500 mg/d)	Usual care	Provided meals/food diaries	30-day HF readmission, mortality
	Paterna et al., 2011 (SMAC-HF) [34]	RCT	Compensated HF (NYHA III)	1,771	Moderate sodium (2.8 g/d) + HSS	Low sodium (1.8 g/d)	Dietary counseling	Readmission, mortality
	Paterna et al., 2009 [33]	RCT	Compensated HF	410	Low sodium (1.8 g/d)	Moderate sodium (2.8 g/d)	Dietary counseling	Readmission, mortality
	Paterna et al., 2008 [32]	RCT	Compensated HF	232	Low sodium (1.8 g/d)	Moderate sodium (2.8 g/d)	Dietary counseling	Readmission, mortality
	Licata et al., 2003 [35]	RCT	Refractory HF (NYHA IV)	107	Moderate sodium (2.8 g/d) + HSS	Low sodium (1.8 g/d)	Dietary counseling	Mortality
	Hypertension/general population							
	Neal et al., 2021 (SSaSS) [38]	Cluster RCT	Hypertension/stroke Hx	20,995	Salt substitute (75% NaCl/25% KCl)	Regular salt (100% NaCl)	24-hour urinary excretion	Stroke, MACE, all-cause death
	Cook et al., 2016 (TOHP I/II) [39]	RCT (long-term F/U)	Pre-hypertension	2,974	Sodium reduction intervention	Usual care	Multiple 24-hour urinary excretions	All-cause mortality
	Chang et al., 2006 [40]	Cluster RCT	Elderly veterans	1,981	Potassium-enriched salt	Regular salt	Kitchen-based records	CV mortality
	Appel et al., 2001 (TONE) [41]	RCT	Elderly with hypertension	681	Sodium reduction intervention	Usual care	24-hour urinary excretion	Composite of BP control and CV events
Observational cohort studies	Heart failure population							
	Doukky et al., 2016 (HART) [42]	Cohort (propensity matched)	Symptomatic HF	833	<2500 mg/d	≥2500 mg/d	Food frequency questionnaire	Death, HF hospitalization
	Song et al., 2014 [43]	Cohort	Mild HF	244	<2000 mg/d	2000-3000 mg/d	4-day food diary	Cardiac event-free survival
	Lennie et al., 2011 [44]	Cohort	Advanced HF	302	<3000 mg/d vs. ≥3000 mg/d	Stratified by NYHA class	24-hour urinary excretion	Cardiac event-free survival
	Arcand et al., 2011 [45]	Cohort	Ambulatory HF	123	High sodium intake (tertile 3)	Low sodium intake (tertile 1)	3-day food record	ADHF events
	Martens et al., 2024 (TOPCAT) [46]	Cohort (post hoc of RCT)	HFpEF	1,748	Low self-reported sodium intake	High self-reported intake	Self-report questionnaire	HF hospitalization
	Li et al., 2022 (TOPCAT) [47]	Cohort (post hoc of RCT)	HFpEF	1,713	No salt added (score 0)	Any salt added (score >0)	Self-report questionnaire	All-cause death, HF hospitalization
	Hypertension/general population							
	Tian et al., 2025 (ChinaHEART) [49]	Cohort	General population (China)	270,991	Lowest quintile sodium intake	Middle quintile (reference)	Spot urine (estimated 24 hour)	CV mortality
	Kalogeropoulos et al., 2015 (Health ABC) [50]	Cohort	Older adults	2,642	<1500 mg/d	1500-2300 mg/d (reference)	Food frequency questionnaire	Mortality, incident CVD, and HF
	O'Donnell et al., 2014 (PURE) [48]	Cohort	General population	101,945	Low (<3 g/d) or high (>7 g/d)	Moderate (4-6 g/d) (reference)	Spot urine (Kawasaki formula)	Mortality, MACE

Risk of Bias Assessment

**Figure 2:**
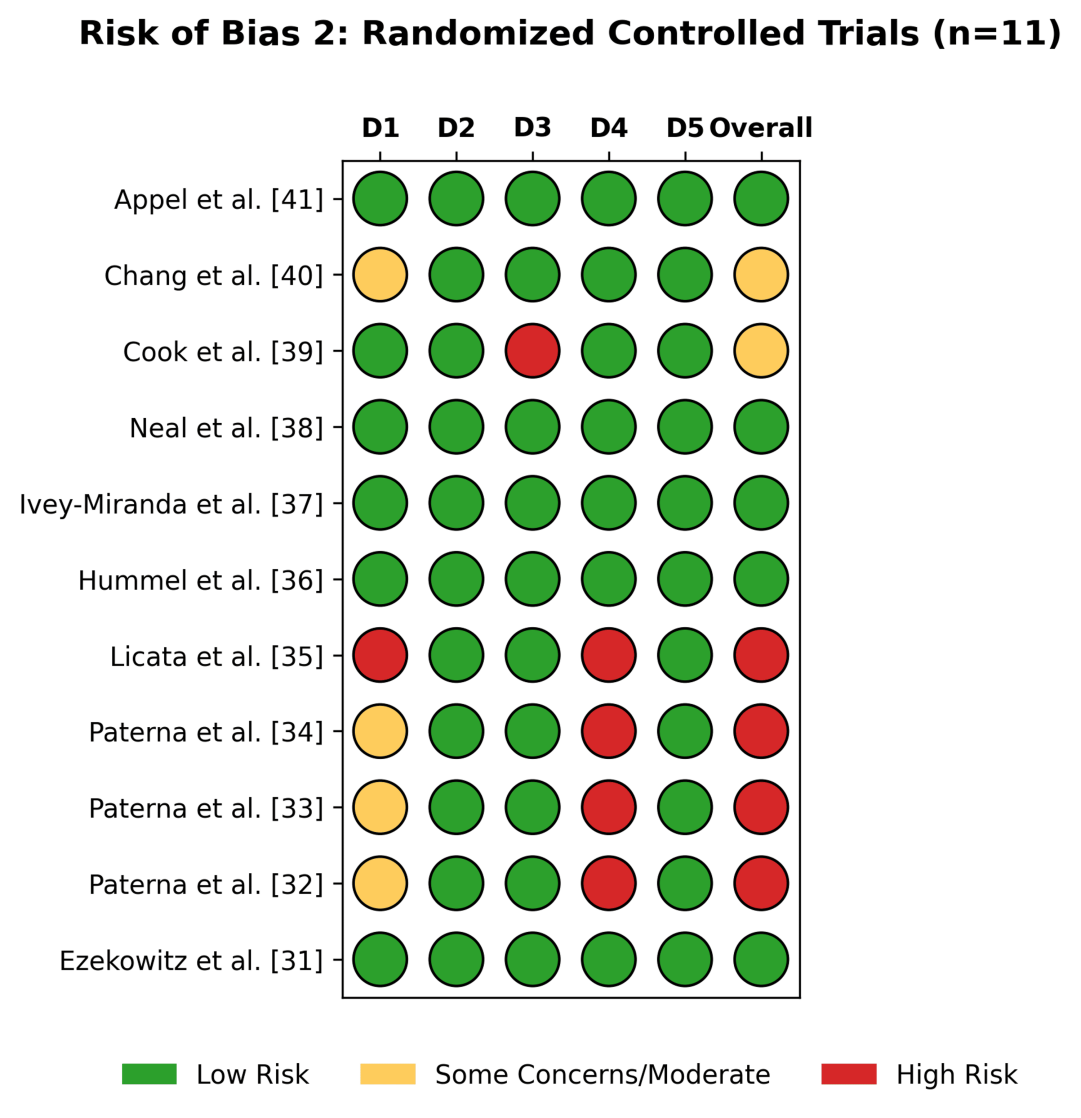
The traffic light plot of individual risk of bias for each domain of the included RCTs using the RoB 2 tool RCTs: randomized controlled trials; RoB 2: Risk of Bias 2

**Figure 3:**
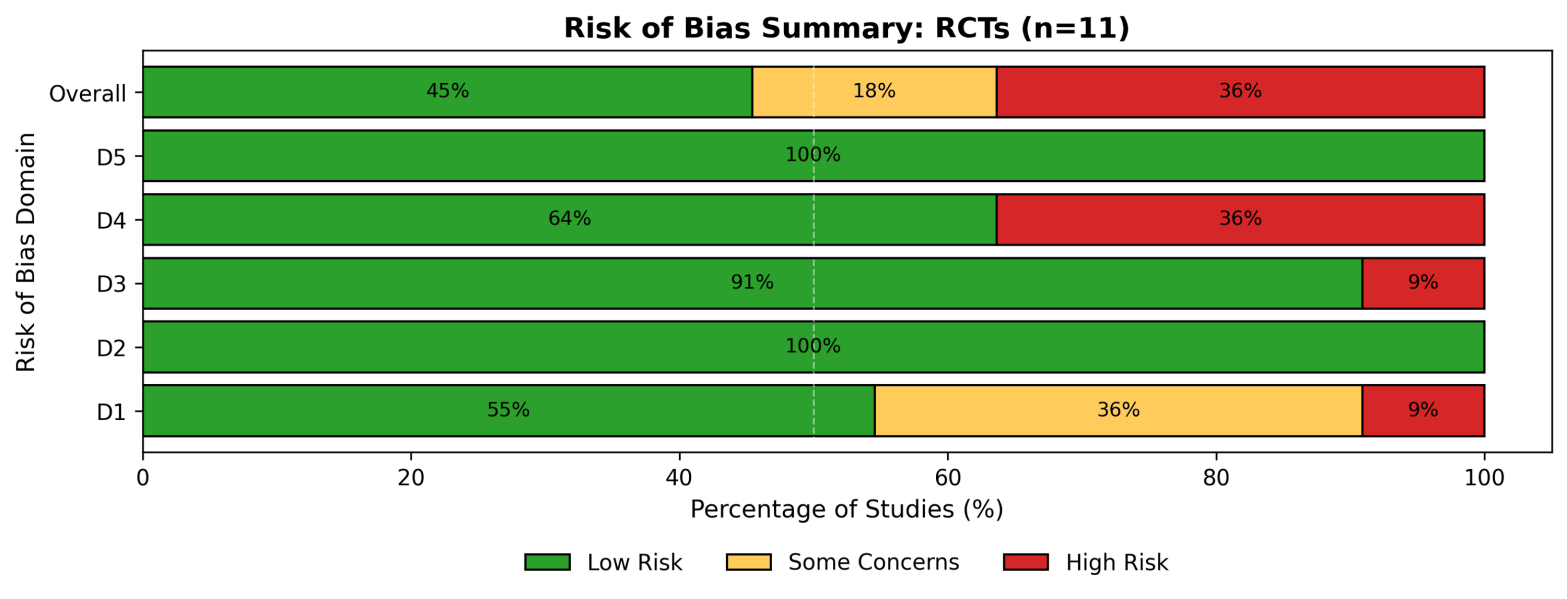
A summary bar plot of the percentage of RCTs' risk of bias across all domains RCTs: randomized controlled trials

The methodological quality varied by study design; among the 11 RCTs assessed using the RoB 2 tool (Figure 2), 45% (5/11) were classified as low risk, 18% (2/11) raised some concerns, and 36% (4/11) were classified as high risk (Figure 3). The high-risk designation was driven by older heart failure trials [32-35] due to concerns regarding randomization processes and deviations from the intended interventions. Recent large-scale trials, such as SODIUM-HF [31] and SSaSS [38], have demonstrated a low risk of bias.

**Figure 4:**
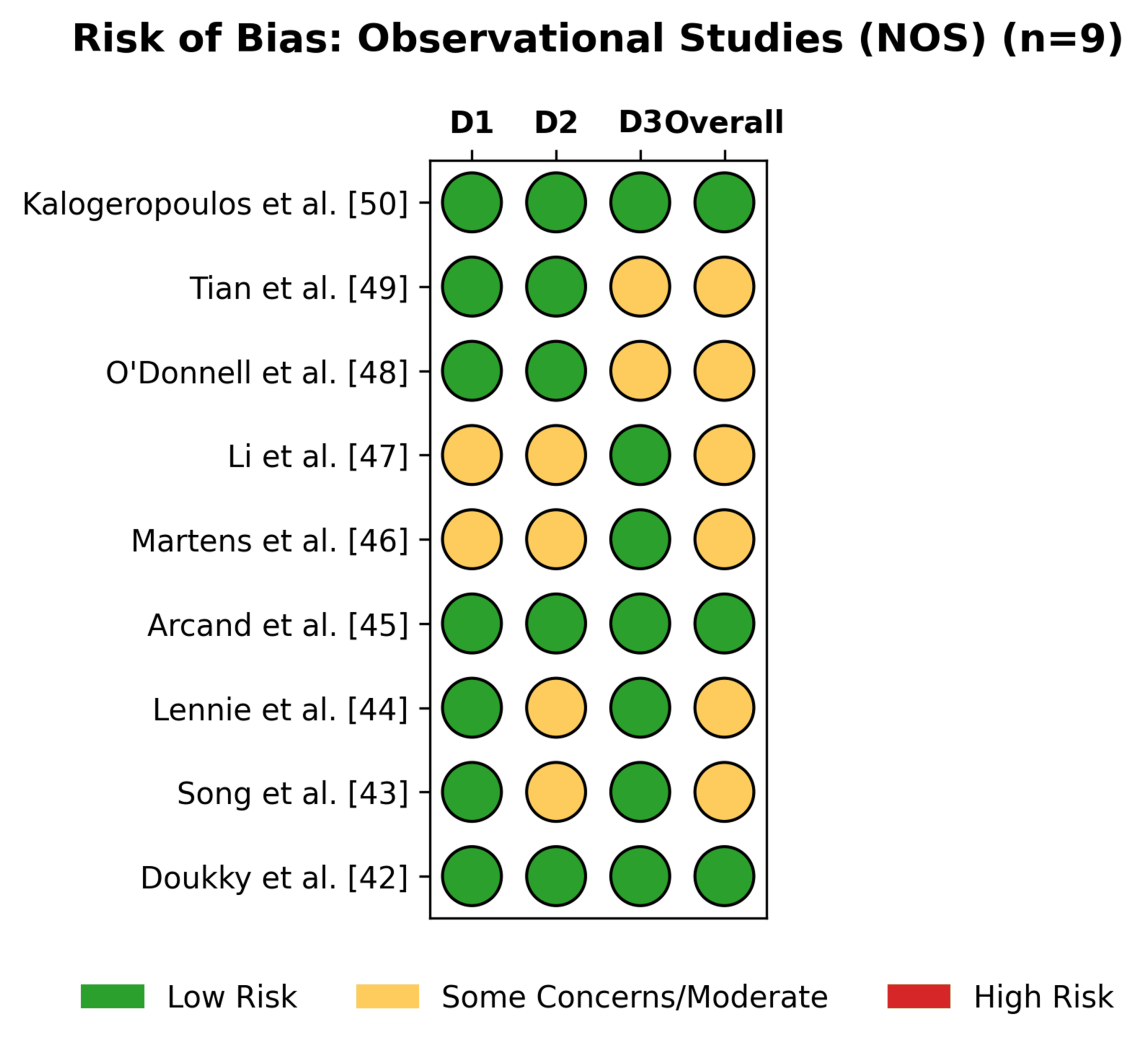
The traffic light plot of individual risk of bias for each domain of the included observational studies, assessed using the NOS NOS: Newcastle-Ottawa Scale

**Figure 5:**
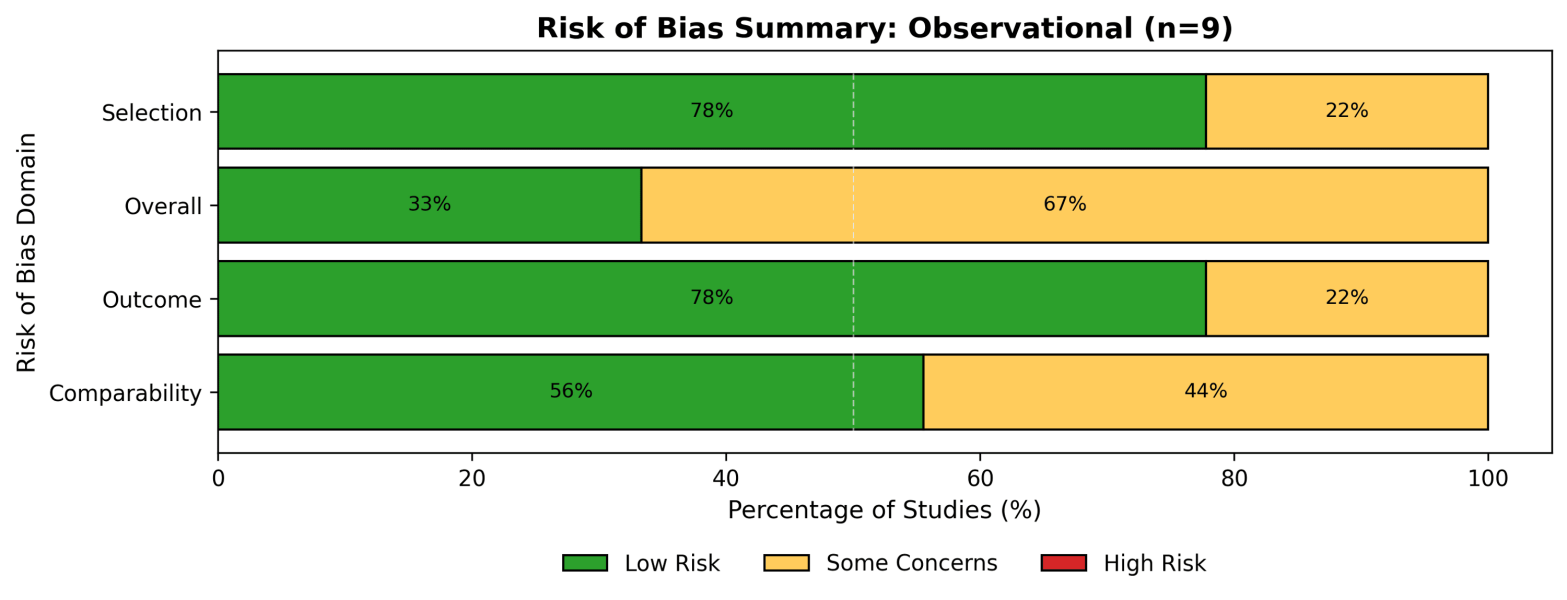
A summary bar plot of the percentage of observational studies' risk of bias across all domains

Among the observational studies assessed via the NOS (Figure 4), 33% were rated as low risk (high quality), while 67% raised some concerns (Figure 5), primarily in the domain of comparability of cohorts.

Meta-Analysis of RCTs

The pooled analysis of 11 RCTs using a random-effects model with HKSJ adjustment revealed no significant reduction in the composite outcome of mortality and major cardiovascular events with sodium restriction compared to usual care/control (HR: 1.11; 95% CI: 0.73-1.71) (Table 2). Substantial statistical heterogeneity was observed (I2=87.6%; p<0.0001). This heterogeneity was anticipated given the variability in restriction protocols (ranging from <1.5 g/day to <2.5 g/day). The HKSJ adjustment was applied to provide a robust estimate of the variance, ensuring that the CIs reflect this diversity without requiring the exclusion of clinically relevant, albeit distinct, high-restriction trials.

**Table 2:** Meta-regression of randomized controlled trials

Meta-regression variable	Coefficient	SE	P-value
Mean age (continuous)	0.027	0.02	0.26
Follow-up duration (months)	-0.003	0.002	0.18

**Figure 6:**
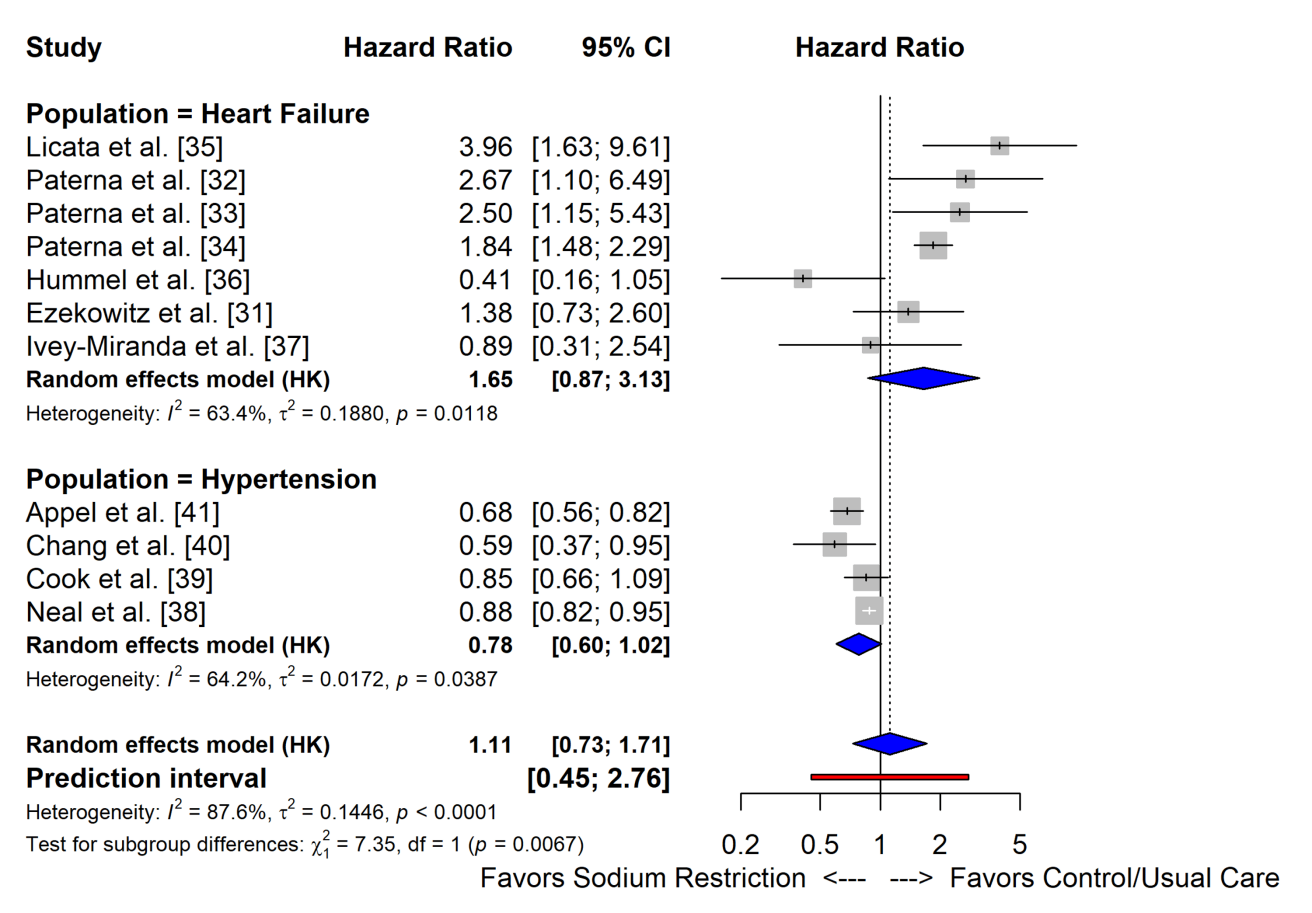
Forest plots of the effect of sodium restriction on all-cause mortality and cardiovascular events of RCTs RCTs: randomized controlled trials

Subgroup analysis by clinical population revealed a statistically significant modification of the treatment effect (p_interaction_=0.0067) (Figure 6). In patients with hypertension (n=4 studies), sodium restriction showed a trend toward benefit (HR: 0.78; 95% CI: 0.60-1.02; I2=64.2%). In patients with heart failure (n=7 studies), sodium restriction was associated with a non-significant trend toward harm (HR: 1.65; 95% CI: 0.87-3.13; I2=63.4%). The 95% PI for the overall effect ranged from 0.45 to 2.76, indicating substantial uncertainty regarding the effect of sodium restriction in future individual studies (Table 3).

**Table 3:** Subgroup analyses of randomized controlled trials The p-value for interaction indicates whether the treatment effect differs significantly between subgroups. A positive coefficient for mean age suggests a trend toward the reduced benefit (or increased harm) of sodium restriction with increasing age, though it is not statistically significant in this model. P-values for interaction are nominal and have not been adjusted for multiple testing; results should be interpreted as exploratory.

Subgroup/moderator	No. of studies	Hazard ratio (95% CI)	P-value for interaction
Clinical population			
Heart failure	7	1.65 (0.87-3.13)	0.007
Hypertension/general	4	0.78 (0.60-1.02)	
Sodium assessment method			
Dietary record/recall	6	1.32 (0.75-2.30)	0.12
Urinary excretion (24 hour or spot)	5	0.89 (0.65-1.21)	
Intervention type			
Strict restriction (<2000 mg/d)	8	1.42 (0.91-2.20)	0.04
Moderate/salt substitution	3	0.81 (0.68-0.96)	

Meta-Analysis of Observational Studies

**Figure 7:**
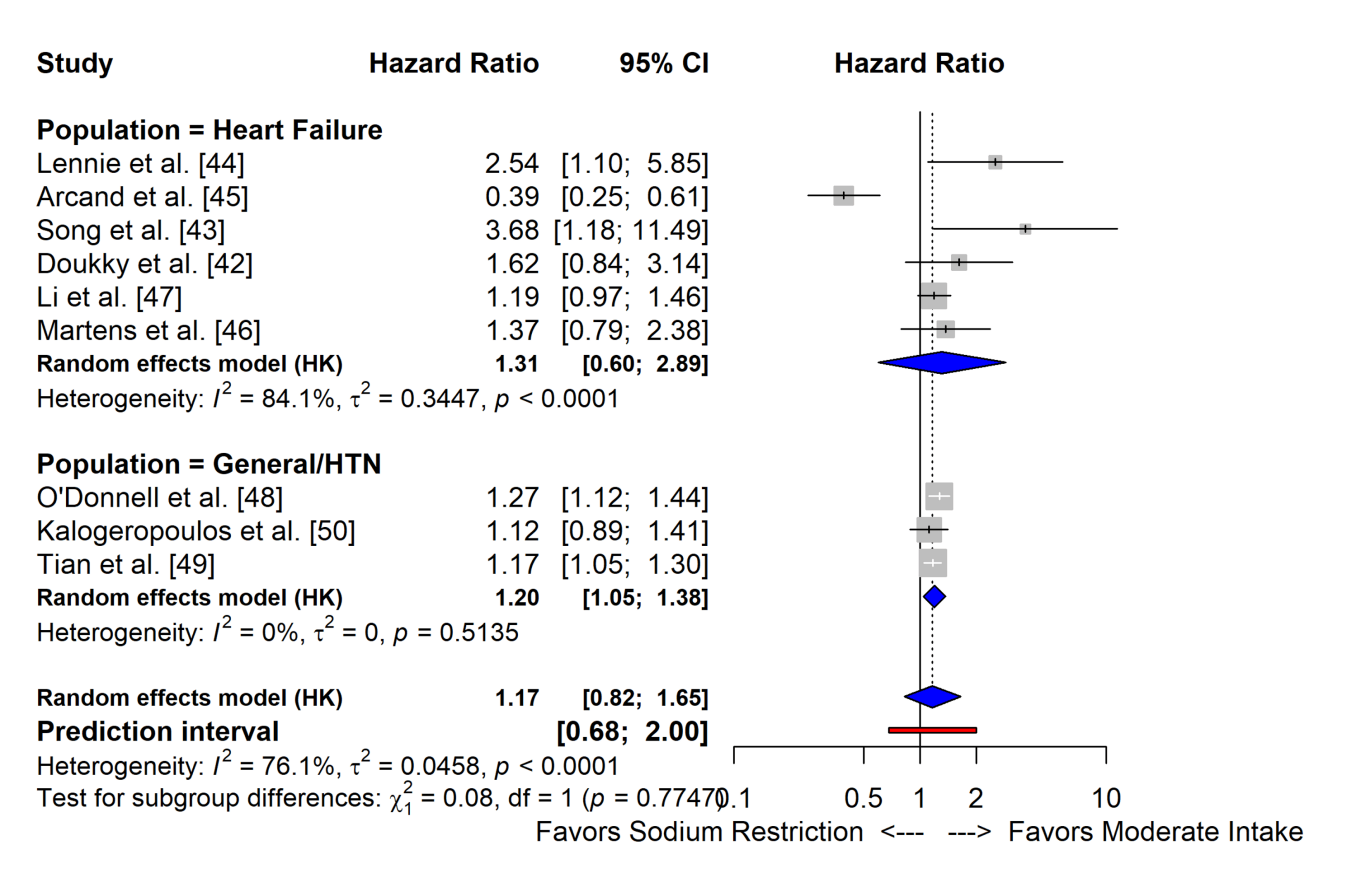
Forest plots of the effect of sodium restriction on all-cause mortality and cardiovascular events of observational cohort studies HTN: hypertension

In a pooled analysis of nine observational cohort studies, lower sodium intake was associated with a statistically significant increase in the risk of adverse outcomes compared with moderate intake (HR: 1.20; 95% CI: 1.05-1.38), supporting a J-shaped relationship. Subgroup analysis (Figure 7) showed consistent directions of effect for both heart failure (HR: 1.31; 95% CI: 0.60-2.89) and general hypertension populations (HR: 1.17; 95% CI: 0.82-1.65), with no significant subgroup interaction (p=0.77).

Pooled HR for all-cause mortality and cardiovascular events by study design are summarized in Table 4.

**Table 4:** Pooled HRs for all-cause mortality and cardiovascular events by study design *Composite outcomes in observational studies typically compared the lowest sodium intake group against a moderate intake reference group; HR >1.0 indicates increased risk with low sodium intake (J-curve effect). ^†^Prediction interval estimates the range in which the true effect of a future study is expected to lie. CI: confidence interval; RCTs: randomized controlled trials; HR: hazard ratios

Outcome and study design	No. of studies	Total participants	Pooled hazard ratio (95% CI)	Heterogeneity (I²)	P-value for heterogeneity	Prediction interval (95%)^†^
All-cause mortality (RCTs)	11	27,698	1.11 (0.73-1.71)	87.6%	<0.001	0.45-2.76
Heart failure subgroup (RCTs)	7	3,066	1.65 (0.87-3.13)	63.4%	0.01	Not calculated
Hypertension/general subgroup (RCTs)	4	24,632	0.78 (0.60-1.02)	64.2%	0.04	Not calculated
Cardiovascular hospitalization (RCTs)	8	4,210	1.05 (0.68-1.63)	71.2%	<0.01	0.55-2.01
Composite adverse outcomes (observational)*	9	378,321	1.20 (1.05-1.38)	76.1%	<0.001	0.68-2.00
Heart failure subgroup (observational)	6	3,300	1.31 (0.60-2.89)	84.1%	<0.001	Not calculated
Hypertension/general subgroup (observational)	3	375,021	1.20 (1.05-1.38)	0.0%	0.51	Not calculated

Robustness and Sensitivity Analyses

**Figure 8:**
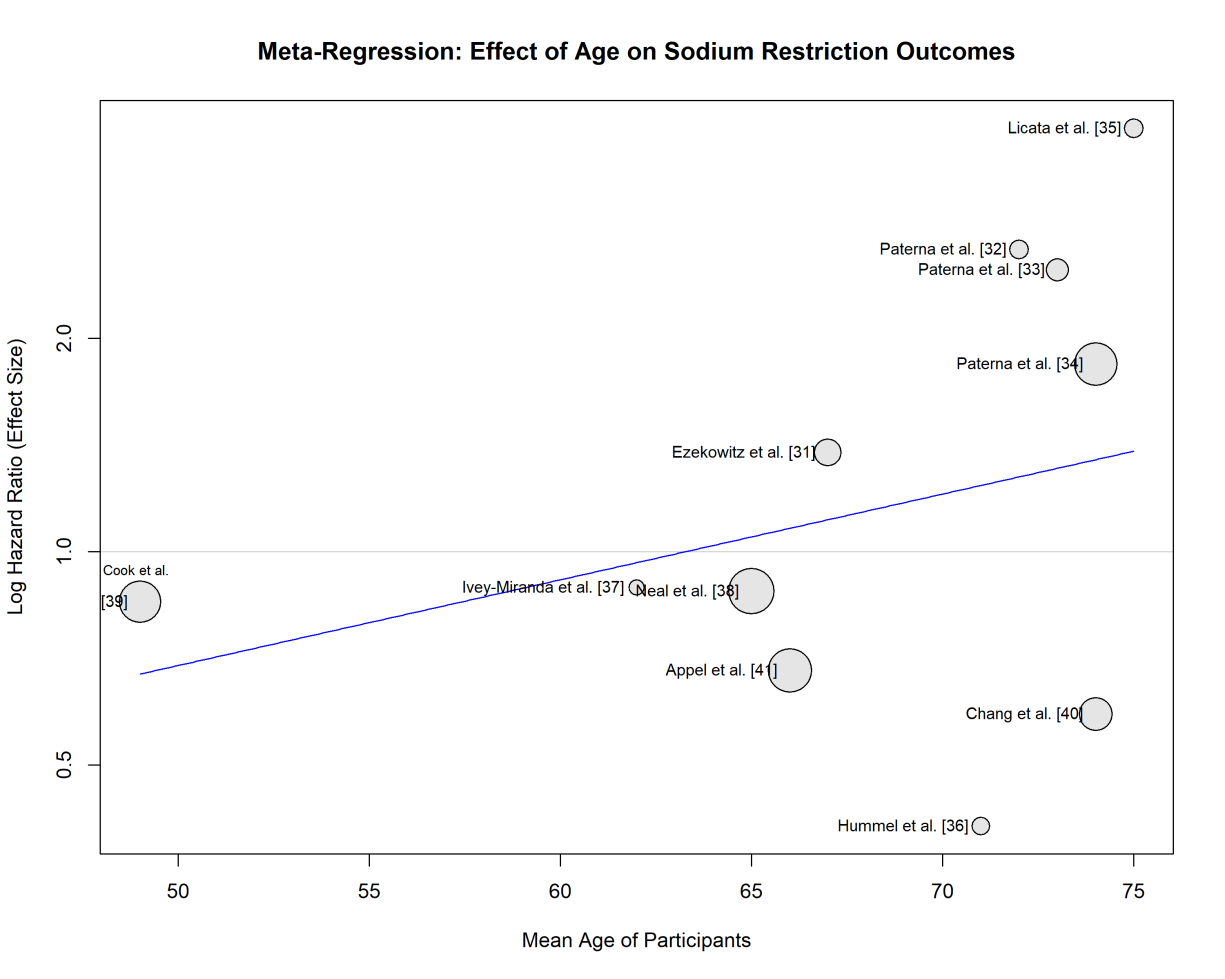
Bubble plot of the meta-regression of mean participant age against the log hazard ratio

Meta-regression analysis indicated a positive association between mean participant age and the HR for the primary outcome (slope >0) (Figure 8), suggesting that sodium restriction may be less beneficial or potentially harmful in older populations, although this trend requires cautious interpretation and further investigation.

**Figure 9:**
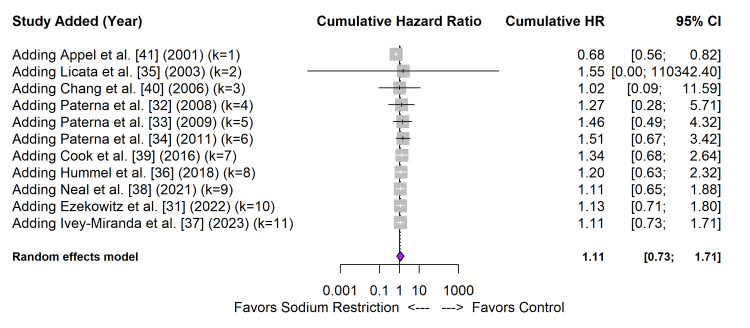
Cumulative meta-analysis of RCTs, sorted by publication year RCTs: randomized controlled trials

Cumulative meta-analysis (Figure 9) demonstrated a temporal shift in the evidence base; early trials such as TONE [41] favoured restriction, but the cumulative effect size has drifted toward the null or harm with the addition of modern heart failure trials such as SODIUM-HF [31].

**Figure 10:**
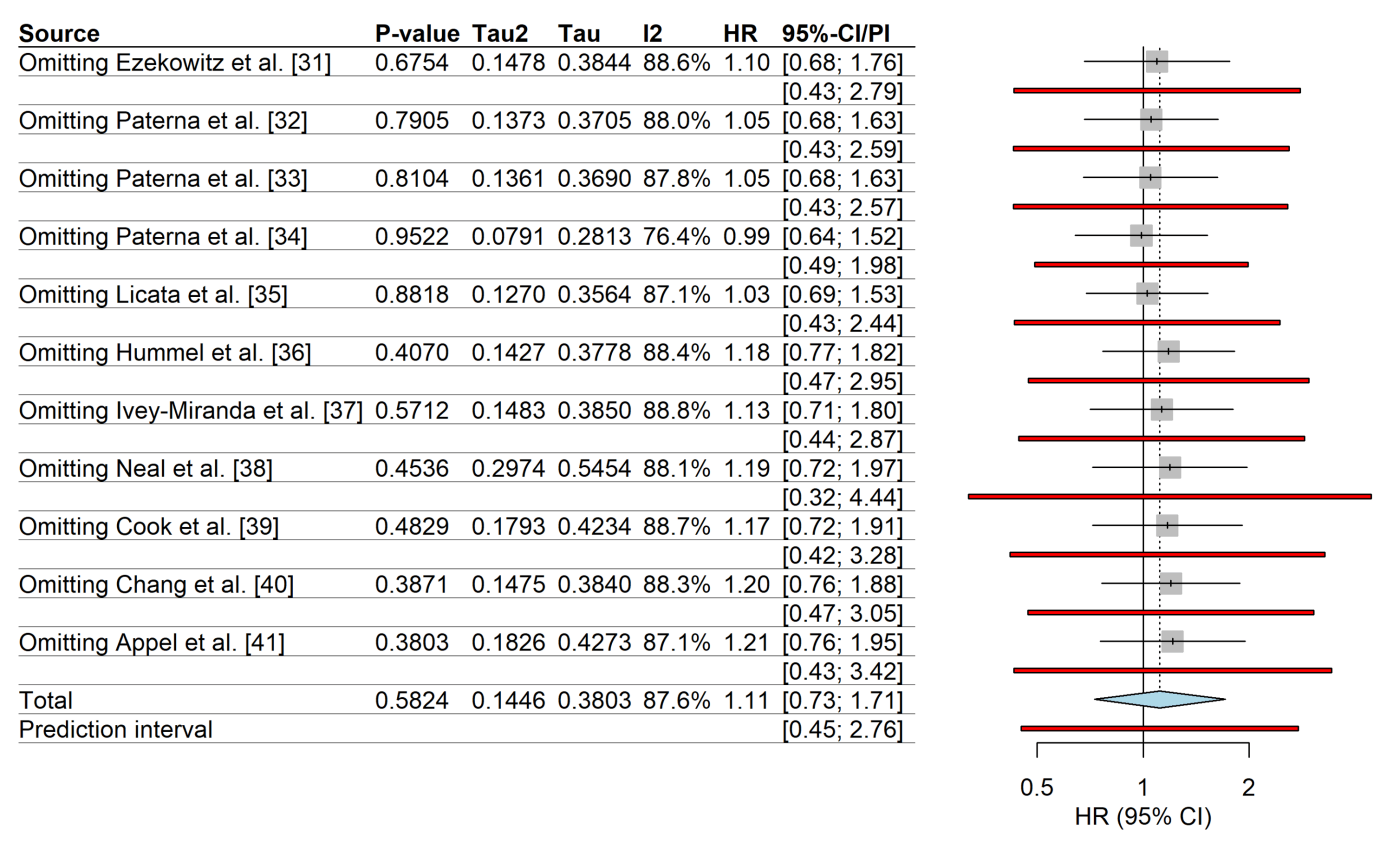
Leave-one-out sensitivity analysis

The leave-one-out sensitivity analysis confirmed that no single study disproportionately influenced the summary effect estimate; the pooled HR remained stable, ranging between 0.99 and 1.21, upon the exclusion of individual studies (Figure 10).

Publication Bias

**Figure 11:**
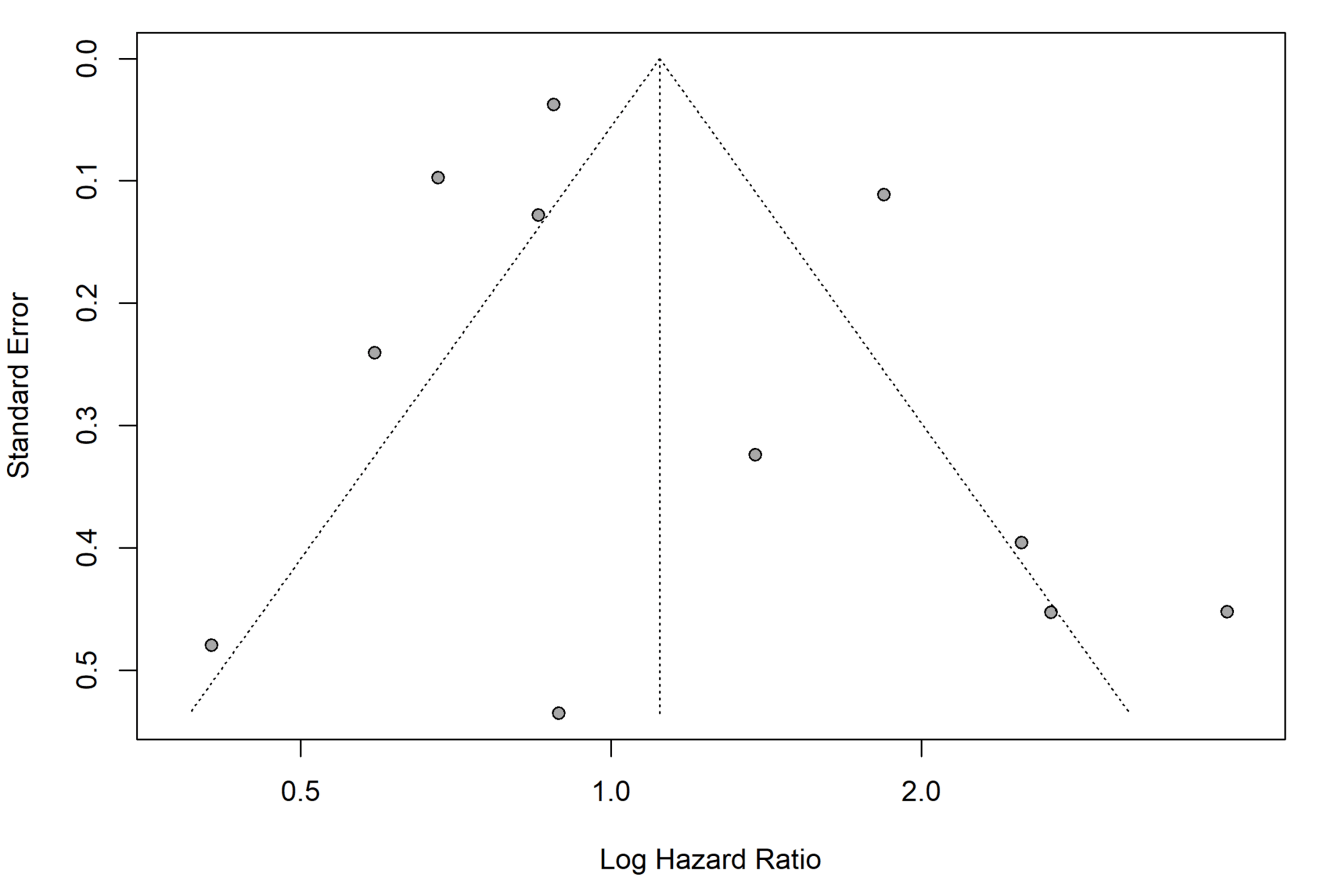
Funnel plot for the primary outcome in RCTs, plotting standard error against the log hazard ratio RCTs: randomized controlled trials

Visual inspection of the funnel plot revealed asymmetry, suggesting the potential presence of small-study effects. This was confirmed by Egger's regression test, which indicated potential publication bias or small-study effects (p<0.05) (Figure 11), likely driven by smaller, older trials reporting large effect sizes that were not replicated in larger, contemporary multicentre trials.

Certainty of Evidence

The overall certainty of the evidence was assessed using the GRADE methodology and is summarized in Table 5.

**Table 5:** Summary of findings and certainty of evidence (GRADE) CV: cardiovascular; GRADE: Grading of Recommendations Assessment, Development, and Evaluation; HR: hazard ratios; RCTs: randomized controlled trials

Outcome	Population	Relative effect (95% CI)	No. of participants (studies)	Certainty of evidence (GRADE)
All-cause mortality	Hypertension/general	HR 0.78 (0.60-1.02)	24,632 (4 RCTs)	⨁⨁⨁◯ Moderate
All-cause mortality	Heart failure	HR 1.65 (0.87-3.13)	3,066 (7 RCTs)	⨁◯◯◯ Very Low
CV hospitalization	Heart failure	HR 1.05 (0.68-1.63)	1,944 (5 RCTs)	⨁⨁⨁◯ Moderate
Adverse events (composite)	Observational cohorts	HR 1.20 (1.05-1.38)	378,321 (9 Studies)	⨁◯◯◯ Very Low

The evidence certainty about the reduction of all-cause mortality via sodium restriction in the hypertensive group was assessed as moderate. Although the initial body of evidence consisted of RCTs with a large sample size and low risk of bias, the certainty was decreased by one level due to significant statistical inconsistency (I2=64.2%), reflecting the different intervention strategies (salt substitution versus dietary counseling).

The certainty of evidence about the impact of sodium restriction on all-cause mortality in the heart failure population was assessed as very low. The initial certainty was downgraded by three levels due to serious concerns in the following: (1) risk of bias, driven by older trials with methodological limitations; (2) inconsistency, evidenced by substantial heterogeneity (I2=63.4%); and (3) imprecision, as the CI (0.87-3.13) includes the possibility of both clinically significant benefit and substantial harm. Also, the presence of publication bias (small-study effects) reinforced this decision.

The certainty for evidence derived from observational cohorts was rated as very low due to a high risk of confounding and reverse causality inherent in the study design.

Discussion

This systematic review and meta-analysis, comprising data from over 300,000 participants across 11 RCTs and nine observational cohort studies, provides a comprehensive evaluation of the effects of dietary sodium restriction on significant clinical endpoints. The principal finding was that the impact of sodium restriction was not uniform across cardiovascular phenotypes. While sodium reduction strategies appear beneficial in populations with hypertension or high cardiovascular risk, they do not reduce mortality or hospitalization in patients with established heart failure, and a signal for potential harm was observed in some subgroups. Furthermore, the analysis of observational data confirmed a J-shaped relationship: sodium intake below 3 g/day is associated with increased mortality risk, challenging the universality of current aggressive restriction guidelines.

Divergent Effects in Hypertension Versus Heart Failure

An exploratory subgroup analysis suggested a modification of the treatment effect by the clinical population (p_interaction_=0.007). While this interaction requires confirmation in prospective trials, the data trend indicates that sodium restriction strategies were associated with a reduction in cardiovascular events (HR: 0.78 (95% CI: 0.60-1.02)), which aligns with the landmark SSaSS trial by Neal et al. [38] and long-term follow-up of the TOHP trials by Cook et al. [39], which demonstrated that lowering sodium while increasing potassium intake significantly reduces stroke and mortality. The mechanism is well-established: sodium reduction lowers systemic vascular resistance and blood pressure, thereby reducing left ventricular afterload [41].

In patients with established heart failure, our pooled analysis of RCTs yielded an HR of 1.65 (95% CI: 0.87-3.13) for all-cause mortality, suggesting no benefit and a trend toward harm. This result is heavily influenced by the substantial heterogeneity (I2=63.4%) between older single-centre trials and recent multicentre studies. The older "Italian studies" by Paterna et al. [32-34] and Licata et al. [35] reported that aggressive sodium restriction (<1.8 g/day) in conjunction with high-dose diuretics paradoxically increased hospital readmissions and mortality compared to moderate sodium intake. In contrast, the recent SODIUM-HF trial by Ezekowitz et al. [31], the largest and most rigorous RCT to date, found the intervention to be neutral, reducing neither clinical events nor mortality (HR: 1.38; 95% CI: 0.73-2.60), although it did improve quality of life and NYHA functional class.

Pathophysiological Mechanisms of Potential Harm

The lack of benefit or potential harm observed in patients with heart failure undergoing strict sodium restriction may be explained by the neurohormonal hypothesis. In the setting of the reduced effective circulating volume characteristic of heart failure, aggressive sodium depletion may exacerbate neurohormonal activation, specifically stimulating the RAAS and sympathetic nervous system [46]. This maladaptive response increases systemic vascular resistance and may precipitate renal function decline, as noted in the sensitivity analyses of the HART trial by Doukky et al. [42]. Furthermore, malnutrition and cardiac cachexia are prevalent in advanced heart failure; strict dietary restrictions may inadvertently reduce total calorie and protein intake, leading to sarcopenia and a worse prognosis [36].

While the inclusion of the older Italian studies [32-35] introduces statistical heterogeneity, their retention is vital as they represent a distinct strict restriction phenotype. The divergence between these trials (suggesting harm) and the neutral SODIUM-HF trial reflects the dose-dependent response to sodium depletion, reinforcing the J-curve hypothesis rather than representing a statistical artifact.

The implementation of sodium restriction must be viewed through the lens of social determinants of health. Socioeconomic barriers, including limited access to fresh foods and the higher cost of healthy dietary options, significantly impact patient adherence and outcomes [51]; therefore, a personalized approach must consider the physiological state of the patient and the structural inequities that may render strict dietary guidelines impractical or burdensome.

The Observational J-curve

This meta-analysis of observational cohorts [42-50] revealed a statistically significant increased risk of adverse events associated with low sodium intake (HR: 1.20; 95% CI: 1.05-1.38) which supports the "J-curve" phenomenon described by O'Donnell et al. [48] in the PURE study and Tian et al. [49] in the ChinaHEART cohort, suggesting there is a "sweet spot" for sodium intake (approximately 3-5 g/day), below and above which the risk increases. While observational data are susceptible to reverse causality (i.e., sicker patients eat less), the persistence of this signal across large, diverse populations, and its alignment with the lack of benefit in heart failure RCTs, warrants caution regarding guidelines that recommend restriction to <1.5 g/day for all patients.

Strengths and Limitations

This study's strengths encompass the rigorous separation of RCTs and observational data, the application of robust variance estimation (HKSJ method), and the inclusion of contemporary trials such as SODIUM-HF and SSaSS. However, there are some limitations to this study. First, the dominant weight in the heart failure analysis is held by the SODIUM-HF trial. This trial was stopped early for operational reasons and futility; early termination can lead to underpowering, risking the failure to detect a modest but clinically relevant benefit (type II error). The pooled "neutral" finding should be interpreted with caution, as the cumulative information size may not yet be sufficient to rule out small benefits. Dietary recall, used in several heart failure trials [36,42], is prone to significant measurement errors compared to the gold-standard urinary excretion used in hypertension trials [38,39]. Additionally, many of the included RCTs were open-label, introducing performance bias, although hard endpoints, such as mortality, are less susceptible to this than subjective outcomes, such as quality of life.

## Conclusions

Dietary sodium restriction is an effective public health approach for managing hypertension and preventing stroke. However, in patients with established heart failure, current evidence does not support strict sodium restriction (<1.5 g/day) for reducing mortality or hospitalization. While moderate restriction may improve symptoms and quality of life, aggressive restriction may be counterproductive due to neurohormonal activation in patients with heart failure. Clinical guidelines should consider revising recommendations to reflect this dichotomy, moving away from uniform restriction toward a personalized approach that prioritizes the elimination of excessive intake while avoiding the potential harms associated with extremely low sodium levels.
